# let-7b suppresses apoptosis and autophagy of human mesenchymal stem cells transplanted into ischemia/reperfusion injured heart 7by targeting caspase-3

**DOI:** 10.1186/s13287-015-0134-x

**Published:** 2015-08-22

**Authors:** Onju Ham, Se-Yeon Lee, Chang Youn Lee, Jun-Hee Park, Jiyun Lee, Hyang-Hee Seo, Min-Ji Cha, Eunhyun Choi, Soonhag Kim, Ki-Chul Hwang

**Affiliations:** Catholic Kwandong University International St. Mary’s Hospital, Incheon Metropolitan City, 404-834 Republic of Korea; Department of Integrated Omics for Biomedical Sciences, Graduate School, Yonsei University, Seoul, 120-752 Republic of Korea; Brain Korea 21 PLUS Project for Medical Science, Yonsei University College of Medicine, Seoul, 120-752 Republic of Korea; Institute for Bio-Medical Convergence, College of Medicine, Catholic Kwandong University, Gangneung-si, Gangwon-do 210-701 Republic of Korea

## Abstract

**Introduction:**

Mesenchymal stem cells (MSCs) have therapeutic potential for the repair of myocardial injury. The efficacy of MSC therapy for myocardial regeneration mainly depends on the survival of cells after transplantation into the infarcted heart. In the transplanted regions, reactive oxygen species (ROS) can cause cell death, and this process depends on caspase activation and autophagosome formation.

**Methods:**

A Software TargetScan was utilized to search for microRNAs (miRNAs) that target caspase-3 mRNA. Six candidate miRNAs including let-7b were selected and transfected into human MSCs *in vitro*. Expression of MEK-EKR signal pathways and autophagy-related genes were detected. Using ischemia/reperfusion model (I/R), the effect of MSCs enriched with let-7b was determined after transplantation into infarcted heart area. Miller catheter was used to evaluate cardiac function.

**Results:**

Here, we report that let-7b targets caspase-3 to regulate apoptosis and autophagy in MSCs exposed to ROS. Let-7b-transfected MSCs (let-7b-MSCs) showed high expression of survival-related proteins, including p-MEK, p-ERK and Bcl-2, leading to a decrease in Annexin V/PI- and TUNEL-positive cells under ROS-rich conditions. Moreover, autophagy-related genes, including Atg5, Atg7, Atg12 and beclin-1, were significantly downregulated in let-7b-MSCs. Using a rat model of acute myocardial infarction, we found that intramyocardial injection of let-7b-MSCs markedly enhanced left ventricular (LV) function and microvessel density, in accordance with a reduced infarct size and the expression of caspase-3.

**Conclusions:**

Taken together, these data indicate that let-7b may protect MSCs implanted into infarcted myocardium from apoptosis and autophagy by directly targeting caspase-3 signaling.

## Introduction

Despite continuous improvements in the prevention and treatment of heart disease, ischemic heart disease is the most common cause of mortality worldwide [1]. Ischemic injury to the heart causes various types of cardiomyocyte death such as necrosis, apoptosis, or autophagy. Various treatment methods for ischemic heart disease have been explored, including gene-based, growth factor-based, and cell-based therapies [2]. Mesenchymal stem cells (MSCs) are an attractive source for cell-based therapies and can be used to repair bone [3], cartilage [4], and heart tissue [5]. In fact, over the last decade, transplantation of MSCs has been considered as a therapeutic method for repairing the infarcted region of ischemic hearts [1]. However, the low viability of cells after transplantation has hampered therapeutic efficacy of cell therapy. Thus, identifying apoptosis-related factors and promoting the survival of transplanted MSCs are key goals for improving the utilization of these cells in cell therapy.

Reactive oxygen species (ROS) play an important role as an inducer of cell death pathways, including apoptosis, anoikis, and autophagy, in ischemic hearts [6]. Among these cell death pathways, apoptosis plays an important role in the elimination of unnecessary cells and is induced by the orchestrated activity of caspase family proteins [7]. Caspase family proteins play a critical role in both the intrinsic and extrinsic apoptosis pathways. During apoptosis, caspases initiate a two-step pathway in which initiator caspases (caspase-8, caspase-9, and caspase-10) cleave executioner caspases (caspase-3 and caspase-7) to activate them [8]. Both caspase-8 and caspase-9 activate caspase-3, which in turn cleaves several crucial substrates including the DNA repair enzyme poly(ADP-ribose) polymerase (PARP) [9]. Autophagy, or self-digestion, is also activated in response to stressors such as ischemia/reperfusion (I/R). Substrates, such as aggregated proteins or damaged organelles, are engulfed during autophagy to form autophagosomes [10, 11]. Although autophagy plays a protective role under a physiologic condition by degrading damaged mitochondria and protein aggregates, excessive autophagy under pathologic conditions can lead to organ dysfunction by degrading essential proteins and organelles [6].

MicroRNAs (miRNAs) are small, noncoding, regulatory RNAs composed of 19–22 nucleotides [12]. miRNAs can act post-transcriptionally to either target specific mRNAs for degradation or suppress their translation [12, 13]. Consequently, miRNAs play critical roles in the regulation of multiple biological processes, including development, organogenesis, cell proliferation, cell differentiation, and apoptosis [13]. However, little is known about how miRNAs regulate cell survival. Recent studies have successfully established a functional link between cell survival and a discrete group of survival-regulating miRNAs, including miRNA-1 [14], miRNA-125 [15], miRNA-206 [14], miRNA-210 [16, 17] and miRNA-708 [18]. In addition, miRNA-210 regulates survival via a positive feedback loop during hypoxia [16]. Nevertheless, most studies have focused on cancer cells as a model to study the mechanisms of hypoxia-regulated miRNAs in their endeavor to escape death in the ischemic microenvironment of tumors, and the role of miRNAs in the survival of transplanted MSCs for therapeutic purposes remains largely unknown. Therefore, in the present study, we examined the feasibility of enhancing survival of transplanted MSCs by modulating miRNA that targets key apoptotic molecule caspase-3. We observed that cleaved (activated) caspase and PARP increased after exposure to ROS in MSCs. Furthermore, our data showed that let-7b plays an important role in cell survival, apoptosis, and autophagy in MSCs under oxidative stress. The results of our study suggest that miRNA-mediated fortification of MSCs by enhancing cell survival can be a potential therapeutic approach to treat infarcted heart.

## Materials and methods

### Culture of human MSCs

Human bone marrow-derived mesenchymal stem cells (hMSCs) were purchased from Lonza (Walkersville, MD, USA). hMSCs were cultured according to the manufacturer’s instructions. We used hMSCs at passages 7–10 for experiments, and the cells were cultured in low-glucose Dulbecco's modified Eagle's medium (DMEM; Life Technologies Corporation, Grand Island, NY, USA) containing 10 % fetal bovine serum (FBS; Life Technologies Corporation).

### miRNA mimic transfection

The miRNA mimics, which are synthesized mature miRNAs or negative control miRNAs with random sequence (NC), were purchased from Genolution Pharmaceuticals (Genolution Inc., Seoul, Korea). Either miRNA mimic or NC (100 nM each) was transfected into hMSCs using siLentFect™ Lipid Reagent (Bio-Rad, Hercules, CA, USA) according to the manufacturer’s instructions. After 4 hours of transfection, the medium was changed to 10 % FBS-containing DMEM with 1 % antibiotics.

### Treatment of cells with hydrogen peroxide

The cells were incubated in low-glucose DMEM containing 10 % FBS. Various concentrations (100–750 μM) of hydrogen peroxide (H_2_O_2_; Sigma, St. Louis, MO, USA) were then added to the medium and incubated with the cells for 6 hours.

### Cell viability assay

Cell viability was determined using a WST-8 (2-(2-methoxy-4-nitrophenyl)-3-(4-nitrophenyl)-5-(2,4-disulfophenyl)-2H-tetrazolium) assay kit (CCK-8 assay kit; Dojindo, Kumamoto, Japan). hMSCs were seeded in 96-well plates (Corning Incorporated, Corning, NY, USA) at a density of 5 × 10^3^ cells per well. The cells were transfected with 100 nM let-7b mimics for 4 hours. After transfection for 4 hours, the medium was changed to fresh 10 % DMEM and the cells were incubated for 24 hours. After incubation, cells were treated with varying concentrations of H_2_O_2_ for 6 hours in 10 % DMEM. The cells were then washed twice with medium, and 100 μl CCK-8 reagent was added to each well. The samples were subsequently incubated for 2 hours at 37 °C. The absorbance of the samples was measured at 450 nm against a background control using a microplate reader (Bio-Rad).

### Real-time PCR

MSCs were plated at a density of 1.5 × 10^5^ cells per well using six-well plates. The cells were first transfected with 100 nM let-7b mimics as described above, and then exposed to varying concentrations of H_2_O_2_ for 6 hours in 10 % DMEM. Total RNA was prepared using Trizol® reagent (Sigma). cDNA for real-time PCR was prepared from purified total RNA using reverse transcriptase (Taqman® MicroRNA Reverse Transcriptase Kit; Applied Biosystems, Carlsbad, CA, USA) with specific miRNA primers. U6 served as a control. The following protocol was used for amplification: 95 °C for 10 minutes, followed by 40 cycles of 95 °C for 15 seconds and 60 °C for 60 seconds. The sequence of the human origin let-7b (hsa-let-7b) primer was 5′-UUG GUG UGU UGG AUG AUG GAG U-3′.

For detecting autophagy-related genes, cDNA was synthesized using a Reverse Transcription System (Promega Corporation, Fitchburg, WI, USA) according to the manufacturer’s instructions. One microgram of RNA was reverse-transcribed in a 20 μl reaction (5 mM MgCl_2_, 10 mM Tris–HCl (pH 9.0 at 25 °C), 50 mM KCl, 0.1 % Triton X-100, 1 mM dNTP, 20 U RNase inhibitor, 0.5 μg oligo(dT) primer, and 10 U reverse transcriptase) for 15 minutes at 42 °C, and then ended at 99 °C for 5 minutes. PCR primers were synthesized by Bioneer Corporation (Daejeon, Korea), and the primers used were as follows: GAPDH, 5′-CAT GGG TGT GAA CCA TGA GAA-3′ and 5′-GGT CAT GAG TCC TTC CAC GAT-3′ (133 base pairs (bp)); autophagy-related (ATG)5, 5′-AGC AAC TCT GGA TGG GAT TG-3′ and 5′-AGG TCT TTC AGT CGT TGT CTG-3′ (139 bp); ATG7, 5′-TTT TGC TAT CCT GCC CTC TG-3′ and ′5-GCT GTG ACT CCT TCT GTT TGAC-3′ (142 bp); ATG12, 5′-ACC ATC CAA GGA CTC ATT GAC-3′ and 5′-CCA TCA CTG CCA AAA CAC TC-3′ (142 bp); and beclin 1, 5′-AAG AGG TTG AGA AAG GCG AG-3′ and 5′-TGG GTT TTG ATG GAA TAG GAG C-3′ (111 bp). PCR reactions were run on a LightCycler 480 SYBR Green I Master (Roche, Penzberg, Germany) protocol. PCR conditions were set to 95 °C for 3 minutes; 95 °C for 30 seconds with 40 cycles of denaturation; 55 °C for 30 seconds; 72 °C for 90 seconds; and 72 °C for 10 minutes as the final extension. The threshold cycle (Ct) of each target gene, which was located in the linear amplification phase of the PCR, was measured automatically and normalized to the cycle number of control (U6 and GAPDH for miRNAs and mRNAs, respectively). The relative expression levels of each miRNA or mRNA were measured (^ΔΔ^Ct) and reported as fold induction (^2ΔΔ^Ct).

### Western blotting analysis

hMSCs were plated at a density of 3 × 10^5^ per 60 mm dish. The cells were first transfected with 100 nM let-7b mimics as described above, and then exposed to varying concentrations of H_2_O_2_ for 6 hours in 10 % DMEM. hMSCs were lysed in 1× lysis buffer (Cell Signaling Technology, Beverly, MA, USA) with protease inhibitor (Roche, Basel, Switzerland) and phosphatase inhibitor cocktail (Roche) at 4 °C for 25 minutes. The protein concentrations were determined using a BCA assay (Pierce Biotechnology, Rockford, IL, USA). Equal amount of proteins were subjected to 8 or 10 % SDS-PAGE. Proteins were transferred to polyvinylidene difluoride membranes (PVDF; Millipore, Billerica, MA, USA) at 100 V and 135 mA for 100 minutes. The membranes were blocked with Tris-buffered saline–0.1 % Tween 20 (TBS-T; both Sigma) and 10 % skim milk (BD Science, San Jose, CA, USA) for 1 hour at room temperature or overnight at 4 °C. The blots were then incubated with primary anti-PARP, anti-caspase-3, anti-phospho-mitogen-activated protein kinase (MEK), anti-MEK, anti-p-extracellular signal regulated kinase (ERK), anti-ERK, anti-Bcl-2, anti-Bax, anti-light chain 3 A/B (LC3A/B), and anti-β-actin antibodies for 1 hour at room temperature or overnight at 4 °C. Polyclonal anti-phospho-MEK, anti-MEK, anti-ERK, anti-LC-3IIA/B, and anti-PARP antibodies were obtained from Cell Signaling Technology. Monoclonal anti-phospho-ERK and monoclonal anti-Bcl2 antibodies were obtained from Santa Cruz Biotechnology (Santa Cruz, CA, USA), polyclonal anti-Bax antibody was obtained from Enzo Life Sciences (Ann Arbor, MI, USA), polyclonal anti-caspase-3 antibody was obtained from Millipore, and β-actin antibody was obtained from Sigma. All antibodies were diluted 1:1000 with TBS-T and 5 % nonfat dried milk. The membranes were then washed three times in 1× TBS-T at room temperature and incubated with a horseradish peroxidase-conjugated rabbit or mouse secondary antibody (Santa Cruz Biotechnology). After washing the membrane six times, immunoreactive proteins were detected using an ECL system (Amersham Biosciences, Tokyo, Japan). The images were quantified using ImageJ software.

### Annexin V/propidium iodide staining

hMSCs were cultured in four-well culture dishes (1 × 10^4^ cells per well; Corning Incorporated). The cells were treated with 500 μM H_2_O_2_ for 6 hours with or without prior let-7b transfection. After 6 hours of H_2_O_2_ treatment, the cells were washed with ice-cold phosphate-buffered saline (PBS; Life Technologies Corporation) for 5 minutes and fixed with 4 % formalin (Sigma) for 10 minutes. After blocking with 500 μl Annexin-binding buffer, the cells were stained with Annexin V-fluorescein isothiocyanate (FITC) at room temperature in the dark for 15 minutes. The dishes were washed with ice-cold PBS, stained with propidium iodide Annexin V/PI staining (PI), and diluted with Annexin-binding buffer at room temperature in the dark for 5 minutes. All images of Annexin V/PI-positive cells were detected by laser scanning confocal microscopy (LSM 700; Carl Zeiss, Thornwood, NY USA), and the images were transferred to a computer equipped with ZEN Lite (Carl Zeiss).

### Measurement of caspase-3 activity

Relative caspase-3 activity was determined using a caspase-3 activity assay kit (Roche). In brief, the cultured MSCs (2 × 10^6^) were lysed in 1× DTT (USB Corporation, Cleveland, OH, USA) for 1 minute. Lysates were collected and loaded onto a plate coated with monoclonal caspase-3 antibody. Upon substrate cleavage, free fluorescent AFC (7-amino-4-trifluoromethylcoumarine) was quantified using a microplate reader (Bio-Rad).

### Terminal deoxynucleotidyltransferase-mediated dUTP nick-end labeling assay

A terminal deoxynucleotidyltransferase-mediated dUTP nick-end labeling (TUNEL) assay was performed according to the manufacturer’s instructions (Millipore). hMSCs were plated in a four-well culture dish (1 × 10^4^ cells per well) and treated with 500 μM H_2_O_2_ for 6 hours with or without prior let-7b transfection. After the slides were rinsed with PBS, the cells were fixed with 10 % formaldehyde (Sigma) for 10 minutes. The slides were treated with 3.0 % H_2_O_2_ and TdT enzyme for 1 hour followed by digoxygenin-conjugated nucleotide substrate at 37 °C for 30 minutes. Nuclei were stained with 3,3′-diaminobenzidine (DAB; Vector Laboratories, Burlingame, CA, USA) for 5 minutes, and the slides were counterstained with methyl green (Sigma). Dark-brown-stained nuclei indicated apoptotic cells. The slides were observed by a virtual microscopy (BX51/dot Slide; Olympus, Tokyo, Japan).

### Luciferase assay

The miRNAs targeting caspase-3 were screened based on the TargetScan miRNA-target prediction database [19]. We amplified the 959 base pair 3′ untranslated region (UTR) of human caspase-3, which contains the binding sites for seven different candidate miRNAs, and the 3′ UTR of human caspase-3 was cloned into the pmirGLO vector (Promega Corporation, Fitchburg, WI, USA). HeLa cells (ATCC, Rockville, MD, USA) were plated in 24-well plates (Corning Incorporated) at a density of 2 × 10^4^ cells per well. The pmirGLO vector containing the 3′ UTR of caspase-3 was co-transfected with let-7b mimic or NC using siLentFect™ (Bio-Rad). Luciferase activity was measured 48 hours later using a luminometer and the Dual Luciferase Assay (Promega Corporation) according to the manufacturer's instructions. *Renilla* luciferase was used to normalize the cell number and transfection efficiency.

### Design of a miRNA-detecting molecular beacon

We have previously developed a molecular beacon (MB) to detect miRNA expression in single cells [20]. MBs are oligonucleotide hybridization probes that indicate the presence of specific nucleic acids. We designed a MB to detect the presence of let-7b, which forms a partially double-stranded structure with a longer Cy3 modified sequence (5′-AAC CAC ACA ACC TAC TAC CTC A-3′-Cy3) and a black hole quencher dye 1 (BHQ1) modified shorter sequence (3′-TGA TGG AGT-5′-BHQ1). The longer sequence of the MB was designed to complementarily match the sequence of let-7b (5′-TGA GGT AGT AGG TTG TGT GGT T-3′) so that when MB and let-7b bind to each other, making the fluorophore and the quencher sufficiently separated, fluorescence from the fluorophore can be detected. These oligonucleotides used for the MB were manufactured by Bioneer Corporation. To find the working concentration of the MB, we conducted an in-vitro assay. Varying concentrations of MB (0, 1, 5, 10, 20, 50, and 100 pM) were reacted with 100 nM let-7b mimic in Eppendorf tubes for 1 hour at 37 °C, and the fluorescence intensity of each group was measured (Varioskan Flash; Thermo Scientific, Waltham, MA, USA). miRNA-23 has a completely irrelevant sequence to the let-7b, and thus a MB designed to detect miR-23 served as a NC. Since a denatured MB should emit fluorescence, boiled MB (at 95 °C for 10 minutes) served as a positive control.

### Detection of let-7b using a MB

hMSCs were seeded onto 24-well plates at a density of 2 × 10^4^ cells per well. After transfection of 100 nM let-7b mimic, the cells were incubated with or without 500 μM H_2_O_2_. To detect cellular let-7b, the cells were transfected with 50 pM MB. The fluorescence intensity was measured (Varioskan Flash; Thermo Scientific).

### I/R injury and transplantation of MSCs

I/R injury was induced in male Sprague–Dawley rats (250 ± 30 g; Coretech, Pyeongtaek, Korea) by surgical occlusion of the left anterior descending coronary artery according to previously described procedures [21]. For transplantation, 1 × 10^6^ cells were suspended in 30 μl PBS and injected from the injured region to the border using a Hamilton syringe with a 30-gauge needle. Throughout the operation, the animals were ventilated with 95 % O_2_ and 5 % CO_2_ using a Harvard ventilator (Harvard Apparatus, Holliston, MA, USA). Five animals per group (ligation, NC-MSCs, let-7b-MSCs) were used for morphological and functional analysis.

### Left ventricular catheterization for hemodynamic analysis

Left ventricular catheterization was performed 3 weeks after infarction to assess hemodynamics. A Millar Mikro-tip 2 F pressure-volume transducer (model SPR-838; Millar Instruments, Houston, TX, USA) was introduced into the left ventricle via the right carotid artery under anesthesia. All data were analyzed offline with PVAN 3.5 software (Millar Instruments).

### Histological analysis and determination of fibrosis area

Heart tissues were fixed in 3.7 % buffered formaldehyde and embedded in paraffin. Tissue sections (5 μm thickness) were deparaffinized, dehydrated, and rinsed with PBS. Fibrosis was analyzed by Masson’s trichrome staining kit (Sigma). Antigen retrieval was performed with 10 mM sodium citrate (pH 6.0; Sigma) in a microwave for 10 minutes. The sections were incubated in 3 % H_2_O_2_ to quench endogenous peroxidase activity. The samples were blocked in 2.5 % normal horse serum (Sigma) and subsequently incubated with antibodies including anti-mouse CD31 and anti-rabbit caspase-3. All antibodies were diluted 1:500 with 1.5 % normal horse serum. All antibodies were obtained from Santa Cruz Biotechnology. FITC-conjugated goat anti-mouse IgG (Jackson ImmunoResearch Laboratories, West Grove, PA, USA) and rhodamine-conjugated goat anti-rabbit IgG (Jackson ImmunoResearch Laboratories) were used as secondary antibodies. All images of CD31 and caspase-3 were obtained using laser scanning confocal microscopy (LSM 710; Carl Zeiss, Thornwood, NY, USA) and transferred to a computer equipped with Zen Light Edition (Zeiss) for analysis. The areas are expressed as percentages of the total left ventricle. Fibrosis was also analyzed by Masson’s trichrome staining.

### Experimental ethics policy

All experimental procedures for animal studies were approved by the Committee for the Care and Use of Laboratory Animals at Yonsei University College of Medicine and were performed in accordance with the Committee’s Guidelines and Regulations for Animal Care.

### Statistical analysis

Data are expressed as the mean ± standard error of the mean of at least three independent experiments. Comparisons between more than two groups were performed by one-way analysis of variance using Bonferroni’s correction. P value of less than 0.05 was considered significant.

## Results

### H_2_O_2_-induced apoptosis of hMSCs

**Fig. 1:**
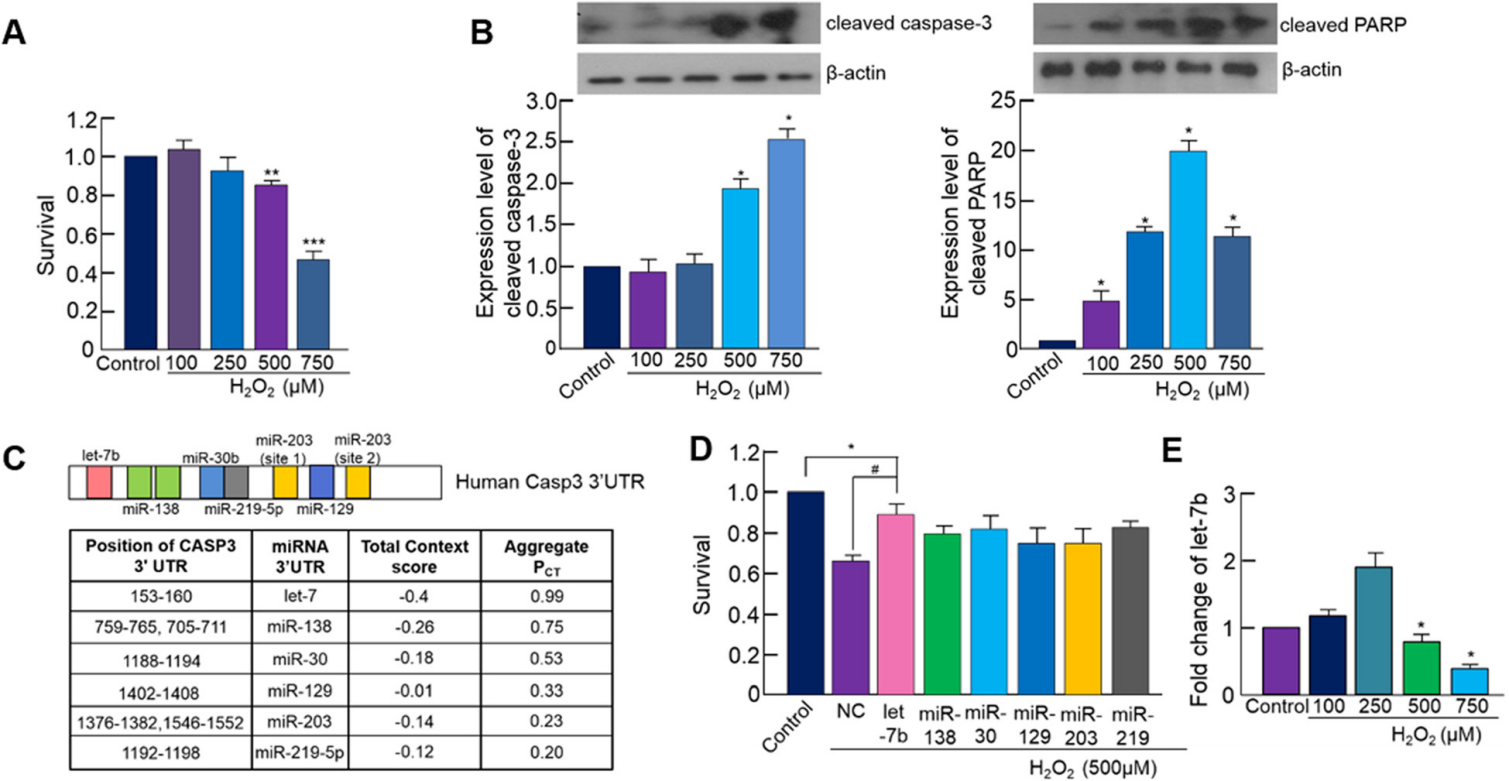
H_2_O_2_-induced apoptosis. **a** Cell survival was evaluated after treating cells with increasing concentrations of H_2_O_2_ for 6 hours (***p <0.0001, **p <0.001). **b** Expression of cleaved caspase-3 and PARP was measured by western blotting in cells treated with increasing concentrations of H_2_O_2_ for 6 hours (*p <0.05). **c** Schematic presentation of miRNA binding site in the 3′ UTR of human caspase-3. Candidate miRNAs predicted to target caspase-3 were selected based on the TargetScan miRNA-target prediction database [19]. miRNAs with an aggregation Pct value ≥0.2 were selected. **d** Cell survival was measured in candidate miRNA-transfected hMSCs. All samples were treated with 500 μM H_2_O_2_ after miRNA transfection. **e** Effect of H_2_O_2_ on endogenous let-7b expression was evaluated by real-time PCR. Quantitative data expressed as mean ± standard deviation of at least three independent experiments. (*p <0.05, and #*p* <0.05)

We used H_2_O_2_ to simulate ROS-mediated cell death in our experiments. To induce apoptosis, hMSCs were treated with varying concentrations of H_2_O_2_. After 6 hours of treatment, cell survival significantly decreased when the concentration of H_2_O_2_ was higher than 500 μM (Fig. 1a). Especially with 500 μM H_2_O_2_ treatment, the amount of both cleaved caspase-3 and PARP also increased (Fig. 1b), suggesting that H_2_O_2_ at this concentration effectively induced apoptosis hMSCs.

### Screening of candidate miRNAs targeting caspase-3

To examine whether miRNA-mediated downregulation of caspase-3 can prevent H2O2-mediated apoptosis, we first searched miRNAs using the TargetScan, miRNA target prediction program [19]. Six candidate miRNAs that are predicted to target caspase-3 (let-7, miR-138, miR-30b, miR-129, miR-203, and miR-219-5p) and have an aggregate Pct greater than 0.2 were selected (Fig. 1c). To empirically validate caspase-3 targeting of those selected miRNAs, the cells were first transfected with each candidate miRNA, and then miRNA transfected cells were exposed to 500 μM of H_2_O_2_ for 6 hours. The cell survival data after miRNA transfection and H_2_O_2_ treatment indicated that let-7b significantly attenuated cell survival after H_2_O_2_ exposure, while other candidate miRNA had no significant effect on cell survival (Fig. 1d). These data suggest that let-7b may exert anti-apoptotic effect. Additionally, H_2_O_2_ treatment significantly decreased the expression of endogenous let-7b at high concentrations (500 and 750 μM) (Fig. 1e), further suggesting that decreased let-7b from H_2_O_2_ treatment may be linked to the decreased cell survival after given concentrations of H_2_O_2_ treatment.

### Inhibition of caspase-3 by let-7b

**Fig. 2:**
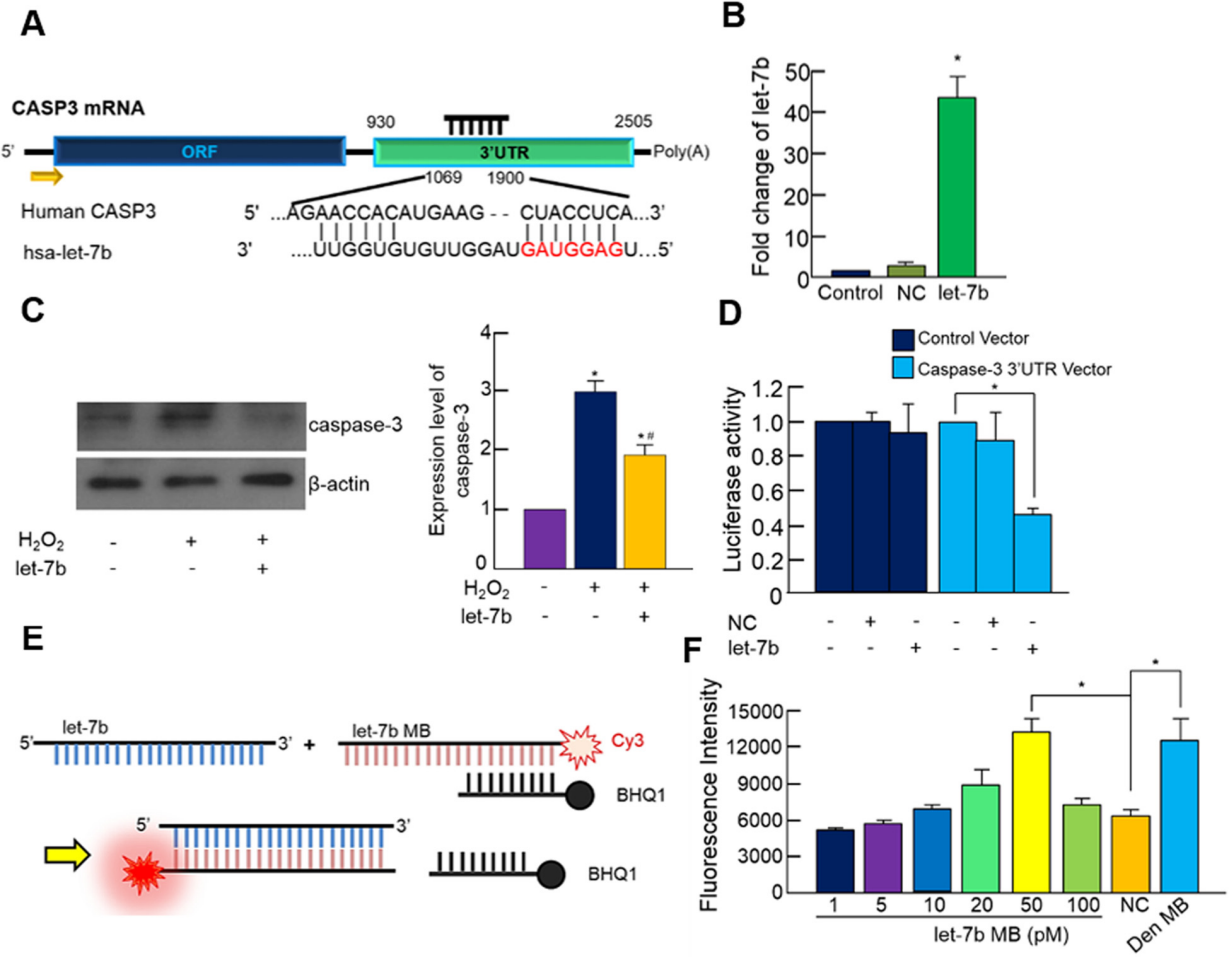
Effect of let-7b on the expression of caspase-3 and design of a molecular beacon (MB) for detecting let-7b. **a** Schematics showing the let-7b binding site in the 3′ UTR of human caspase-3. **b** Efficiency of let-7b transfection was measured by real-time PCR (*p <0.05). **c** Caspase-3 expression with or without let-7b transfection prior to H_2_O_2_ treatment was detected by western blotting (*p <0.05). **d** Let-7b targeting of caspase-3 was evaluated by a luciferase assay using luciferase vector containing the 3′ UTR of human caspase-3. HeLa cells were transfected with control vector or vector containing the 3′ UTR of human caspase-3 along with negative control miRNA (NC) or let-7b mimics (100 nM each) (*p <0.05). **e** Schematics showing the working principle of a MB designed to detect let-7b. **f** Working concentration of the MB was determined by an in-vitro assay using 100 nM let-7b and increasing concentrations of MB designed to detect let-7b. MB designed to detect miR-23 served as NC and denatured MB (Den MB) served as a positive control (*p <0.05). Quantitative data expressed as mean ± standard deviation of at least three independent experiments

Sequence alignment indicated that let-7b recognizes and binds to the sequence between 1069 and 1900 of the caspase-3 3′ UTR (Fig. 2a). When the cells were transfected with 100nM let-7b mimics, the amount of cellular let-7b detected by real-time PCR increased approximately 40-fold compared with nontransfected cells (Fig. 2b). Transfection of let-7b mimics significantly decreased the expression of caspase-3 (Fig. 2c) and the luciferase activity of cells transfected with luciferase vector containing the 3′ UTR of human caspase-3 (Fig. 2d), indicating that let-7b directly targeted caspase-3.

### Visual detection of let-7b in vitro using a MB

We utilized MBs to detect intracellular let-7b. MBs are oligonucleotide hybridization probes that indicate the presence of specific nucleic acids. The Cy3-modified longer sequence of the MB complementarily matches the sequence of let-7b, so that when let-7b binds to the MB it disturbs fluorophore (Cy3)–quencher (BHQ1) interaction producing fluorescence (Fig. 2e). To determine the optimal amount of MB for detecting let-7b, we conducted an in-vitro assay using 100 nM let-7b mimics and increasing concentrations of MB designed to detect let-7b (1–100 pM), and the result indicated that 50pM MB produced the most strong fluorescent signal (Fig. 2f). Additionally, the negative control group (100 pM MB designed to detect miR-23 was used instead of MB for let-7b) did not produce significant fluorescence with the presence of let-7b mimic, suggesting the MB for let-7b was indeed specific for detecting let-7b. Since denatured MB should emit fluorescence, boiled MB (at 95 °C for 10 minutes) served as a positive control.

### Anti-apoptotic effect of let-7b on H_2_O_2_-treated MSCs

**Fig. 3:**
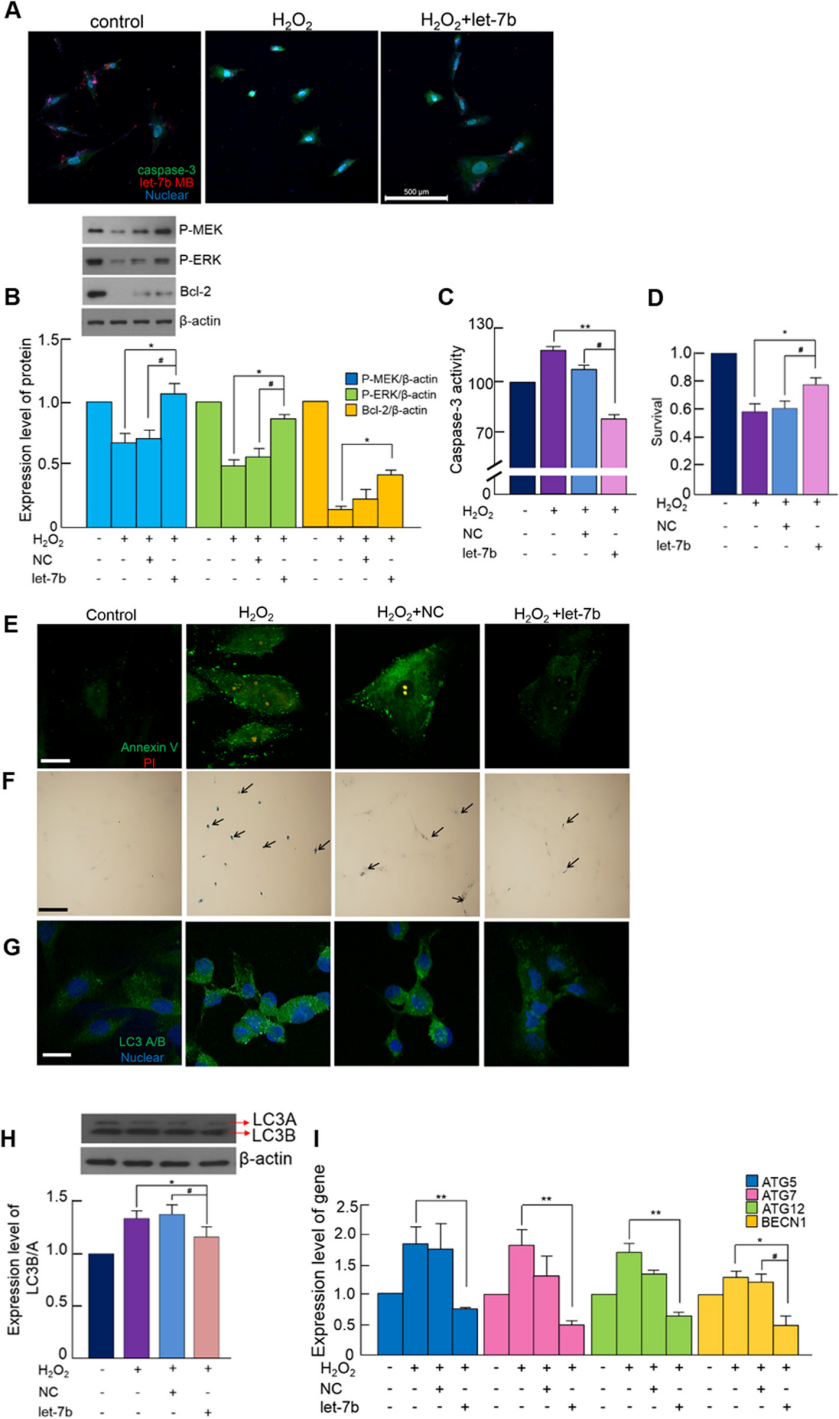
let-7b upregulates survival signaling but downregulates autophagy-related molecules. **a** Intracellular caspase-3 (green) and let-7b (red) were visualized under confocal microscopy. The cells were either untreated (control), H_2_O_2_-treated (H_2_O_2_), or transfected with let-7b prior to H_2_O_2_ treatment (H_2_O_2_ + let-7b). Nuclei stained blue. Scale bar = 500 μm. **b** Various survival-related proteins were detected by western blotting. (*p <0.05, #*p* <0.05). **c** Caspase-3 activity and **d** cell survival was evaluated. (*p <0.05, #*p* <0.05 ). **e** Annexin V (green)/PI (red) staining. **f** TUNEL staining. **g** LC3A/B (green) was detected by confocal microscopy. Nuclei stained blue. Scale bar = 100 μm. **h** Expression of LC3A/B was detected by western blotting (*p <0.05, #*p* <0.05). **i** Expression of autophagy-related genes was evaluated by real-time PCR (**p <0.001, *p <0.05, #*p* <0.05). Quantitative data expressed as the mean ± standard deviation of at least three independent experiments

When the cells were treated with 500 μM H_2_O_2_, the expression of caspase-3 increased while let-7b expression decreased. However, such an increase of caspase-3 and decrease of let-7b were attenuated by let-7b transfection prior to H_2_O_2_ treatment (Fig. 3a). To determine the effect of let-7b on survival-related signaling, we examined the phosphorylation of MEK and ERK and the expression of anti-apoptotic protein Bcl-2. H_2_O_2_ decreased phosphorylation of both MEK and ERK and the expression of Bcl-2. However, this H_2_O_2_-induced decrease was attenuated by let-7b transfection prior to H_2_O_2_ treatment (Fig. 3b). Furthermore, increased caspase-3 activity by H_2_O_2_ treatment was also attenuated by let-7b transfection (Fig. 3c), and cell survival was also recovered by let-7b transfection (Fig. 3d), indicating that let-7b-mediated downregulation of caspase-3 attenuated H_2_O_2_-induced cell death. To further validate the anti-apoptotic effect of let-7b, Annexin V/PI staining and TUNEL assays were performed on H_2_O_2_-treated cells with or without let-7b transfection prior to H_2_O_2_ treatment. Annexin V/PI staining indicated that H_2_O_2_ increased both Annexin V and PI staining of MSCs showing increased apoptosis, but this was attenuated by let-7b transfection prior to H_2_O_2_ treatment (Fig. 3e). Furthermore, the number of TUNEL-positive apoptotic cells increased by H_2_O_2_ treatment, but such an increase was suppressed by let-7b transfection (Fig. 3f), suggesting that the delivery of exogenous let-7b effectively prevented H_2_O_2_-induced apoptosis of MSCs. Previous studies have demonstrated that miRNAs were involved in the regulation of autophagic pathway in various cell types [22, 23]. Thus, we also examined the effect of let-7b on autophagy-related genes. H_2_O_2_ treatment increased the expression of LC3A/B, indicating activation of autophagic pathway. However, such an increase of autophagic activity was attenuated by let-7b transfection prior to H_2_O_2_ treatment, and this was confirmed by both immunocytochemistry (Fig. 3g) and western blot (Fig. 3h). Additionally, the expression of autophagy-related genes, such as ATG5, ATG7, ATG12, and BECN1, was attenuated in the MSCs transfected with let-7b (Fig. 3i). These results indicated that let-7b regulates the expression of autophagy-related genes at both the mRNA and protein levels.

### Let-7b-enriched MSCs restore cardiac function after ischemic injury

**Fig. 4:**
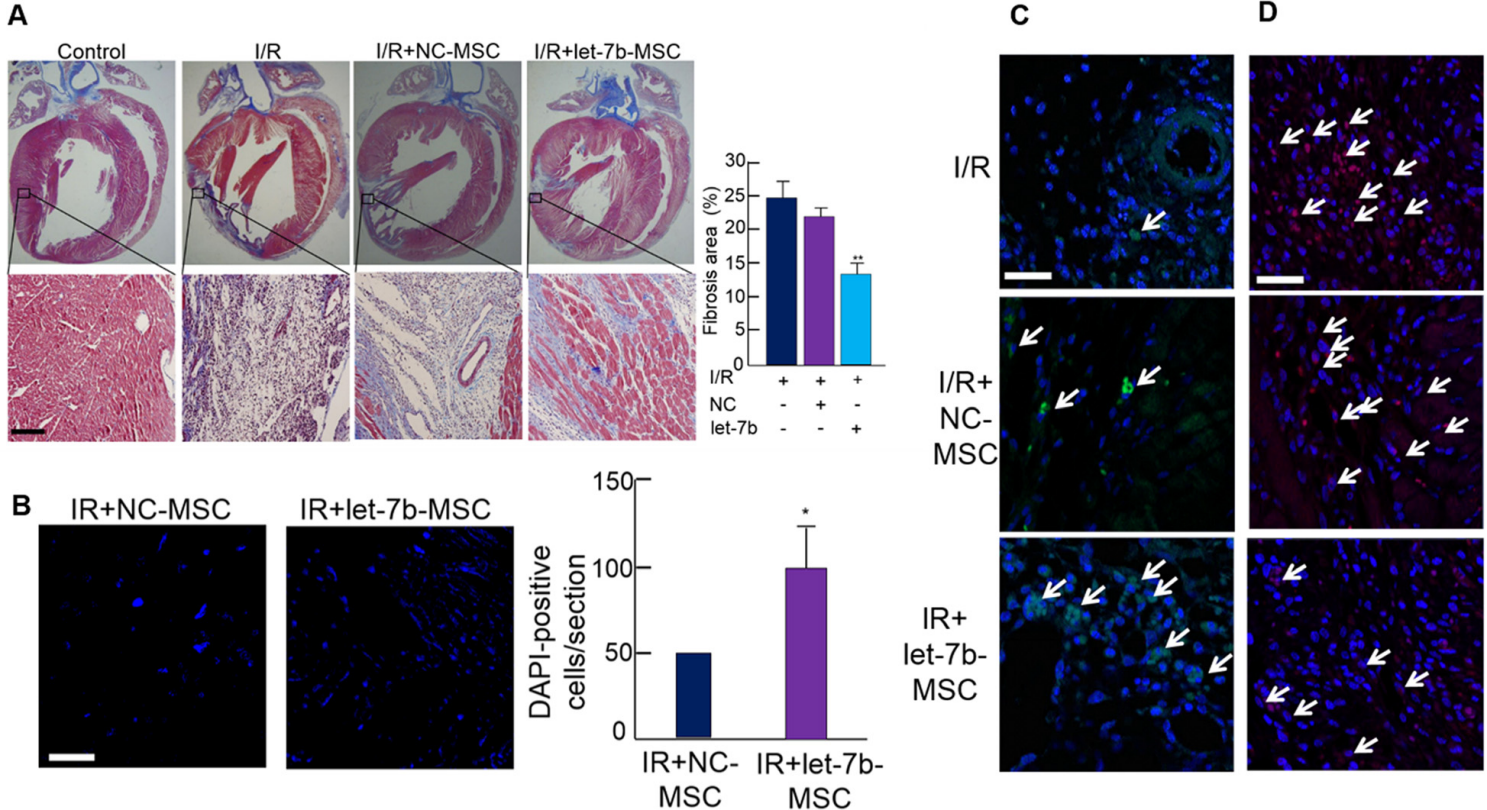
Let-7b-MSCs improved ischemic heart function. **a** Fibrosis area was determined using Masson’s trichrome staining (**p <0.001). **b** To track the transplanted cells, cells were stained with DAPI prior to transplantation. Three days after transplantation, the number of DAPI stained cells was counted (*p <0.05). **c** Angiogenesis was evaluated by CD31 (green, arrow) staining. **d** Caspase-3 (red, arrow) was stained as a marker of apoptosis. Scale bar = 50 μm. Quantitative data expressed as the mean ± standard deviation of at least three independent experiments

To examine the therapeutic potential of let-7b-enriched MSCs in ischemic myocardium, we transplanted let-7b-enriched MSCs into ischemic rat hearts. The area of fibrosis significantly decreased in the hearts transplanted with let-7b-enriched MSCs (let-7b-MSCs) compared with those of untreated ischemic hearts or the hearts transplanted with negative control miRNA-enriched MSCs (NC-MSCs) (Fig. 4a). To assess the survival of transplanted MSCs, we stained the cells with 4′,6-diamidino-2-phenylindole (DAPI) prior to transplantation. The number of DAPI-positive cells was counted 3 days after the transplantation, and the results indicated that the number of DAPI-stained cells increased in the let-7b-MSC transplanted heart compared with the NC-MSC transplanted heart (Fig. 4b). The result of immunohistochemistry using endothelial cell marker CD31 indicated that the transplantation of let-7b-enriched MSCs increased the number of CD31-positive cells per field in infarcted myocardium compared with NC-MSC transplanted heart (Fig. 4c), while the number of caspase-3-positive cells decreased in this group (Fig. 4d), indicating that the transplantation of let-7b-enriched MSCs effectively prevented apoptosis but enhanced angiogenesis in infarcted myocardium. Additionally, analysis of cardiac function indicated that the transplantation of let-7b-enriched MSC improved functional parameters such as stroke volumes, end-diastolic volumes, end-systolic volumes, stroke work, stroke volume, and ejection fraction compared with other groups (Table 1). Taken together, these data suggest that the let-7b-mediated downregulation of caspase-3 is a viable therapeutic strategy for the treatment of ischemic myocardium.

**Table 1:** Effect of let-7b-MSCs on cardiac function in ischemia/reperfusion animal model

	Control	I/R	I/R + MSCs	I/R + let-7b-MSCs
EF (%)	69.68 ± 1.48	40.96 ± 2.42	53.4 ± 0.92*	65.29 ± 1.15**
ESV (μl)	62.67 ± 1.82	120.96 ± 1.95	87.85 ± 1.95*	74.52 ± 1.25**
EDV (μl)	124.43 ± 1.06	189.49 ± 1.95	149.48 ± 2.17*	135.93 ± 2.00*
CO (μl/minute)	37 675.1 ± 2584.66	16 335.9 ± 925.71	24 154.9 ± 589.04	28 580 ± 500.51*
SW (mmHg* μl)	12 710 ± 896	6335 ± 475	10 245 ± 350*	12 526 ± 442*
SV (μl)	156.91 ± 10.57	76.9 ± 5.79	110.14 ± 7.09*	112.68 ± 1.69*

All values expressed as mean ± standard error of the mean

*p <0.05 vs. I/R group

**p <0.001 vs. I/R group

*CO cardiac* output, *EDV* end-diastolic volume, *EF* ejection fraction, *ESV* end-systolic volume,* I/R* ischemia/reperfusion, *MSC* mesenchymal stem cell, *SV* stroke volume, *SW* 1:13 stroke work

## Discussion

MSCs have been used in cell-based therapy for cardiac repair and they are amenable to various pretransplant treatments including cytokines and growth factors, preconditioning, and genetic modification [24]. However, low cell survival after transplantation caused by apoptosis of injected cells due to the loss of matrix attachment in the infarcted region remains a major problem [25]. Consequently, inhibition of apoptosis has long been emphasized in the treatment of heart disease. Apoptosis contributes to the pathogenesis of I/R injury, and apoptosis-related caspase family molecules play important roles in the progression of heart disease [26]. In myocardial infarction, Ca^2+^ overload and ROS can induce apoptosis in the ischemic area [27]. In addition, several studies have reported that autophagy is upregulated in the injured heart [6, 27]. However, it is still unclear whether autophagy plays a protective or detrimental role in the damaged heart. For example, autophagy during energy starvation under mild ischemic conditions delayed irreversible cell injuries, including apoptosis and necrosis [28], while autophagy under harsh conditions, such as I/R which we simulated using an animal model for this study, contributed to cell death via the generation of ROS [27, 29]. Additionally, several published studies have suggested that crosstalk between apoptosis and autophagy exists [30]. The Atg4 family member Atg4D is cleaved by caspase-3, and the expression of beclin-1 regulates the expression of caspase-9 [31, 32]. In the present study, ROS increased the expressions of autophagy-related genes, including Atg5, Atg7, Atg12, and beclin-1, as well as the autophagy marker LC3A/B. However, such ROS-induced increase of autophage-related gene expressions was attenuated by let-7b transfection, and this may have attributed to the increased survival of let-7b transfected cells.

Recently, several studies have demonstrated great potential of miRNAs as a new therapeutic means for ischemic heart disease. A number of miRNAs have been reported to target caspase-3. miR-378 has been reported to attenuate apoptosis of cardiomyocytes by targeting caspase-3 [33]. Furthermore, members of the let-7 family—namely let-7a, let-7e, and let-7 g—have also been reported to target caspase-3 in cancer cells, PC12 cells, and endothelial cells, respectively [26, 34, 35]. Aside from the miRNAs that are not members of the let-7 family, the predicted binding sequence of let-7 family members (a, b, c, d, e, f, g, and i) to the 3′ UTR of human caspase-3 is identical. The only difference was that let-7b had a lower context-positive score (−0.40 for let-7b vs. –0.39 or −0.38 for other members). Since we did not examine the effect of other let-7 family members on caspase-3 expression in the present study, it is difficult to draw any conclusion on the specificity of let-7b in MSCs. This is one of the limitations of the present study, and specific roles of individual let-7 family members on the cell survival, especially of MSCs, will be an interesting subject of further study. Although additional studies are required to further elucidate the underlying mechanisms, our study also demonstrated that modulation of caspase-3 using let-7b can be an effective means to enhance post-transplantation survival of MSCs and subsequent functional recovery of the damaged heart by upregulating survival signals such as MEK and ERK, while suppressing apoptotic signaling activation.

## Conclusions

We have provided an effective strategy for enhancing cell survival after transplantation. Our study indicates that the regulation of caspase-3 by let-7b can improve survival of hMSCs via the modulation of survival signaling and autophagy-related genes. This finding suggests that the compensation of let-7b prior to cell transplantation can be a therapeutically sound approach for treating ischemic hearts.

## Abbreviations

ATG: Autophagy related
BHQ1: Black hole quencher dye 1
bp: Base pairs
Ct: Threshold cycle
DAB: 3,3′-Diaminobenzidine
DAPI: 4′,6-Diamidino-2-phenylindole
DMEM: Dulbecco's modified Eagle's medium
EKR: Extracellular signal regulated kinase
FBS: Fetal bovine serum
FITC: Fluorescein isothiocyanate
H_2_O_2_: Hydrogen peroxide
hMSC: Human bone marrow-derived mesenchymal stem cell
I/R: Ischemia/reperfusion
LC3A/B: Light chain 3 A/B
let-7b-MSC: let-7b-overexpressing MSC
MB: Molecular beacon
MEK: Mitogen-activated protein kinase
MSC: Mesenchymal stem cell
miRNA: MicroRNA
NC: Negative control miRNA
PARP: Poly(ADP-ribose) polymerase
PBS: Phosphate-buffered saline
PI: Propidium iodide
ROS: Reactive oxygen species
TBS-T: Tris-buffered saline–0.1 % Tween 20
TUNEL: Terminal deoxynucleotidyltransferase-mediated dUTP nick-end labeling
UTR: Untranslated region
